# Novel optical soliton molecules formed in a fiber laser with near-zero net cavity dispersion

**DOI:** 10.1038/s41377-023-01074-w

**Published:** 2023-02-07

**Authors:** Xiao Hu, Jun Guo, Jun Wang, Jie Ma, Luming Zhao, Seongwoo Yoo, Dingyuan Tang

**Affiliations:** 1 Julong College, Shenzhen Technology University, 518118 Shenzhen, China; 2 School of Electrical and Electronic Engineering, Nanyang Technological University, Singapore, 639798 Singapore; 3 Jiangsu Key Laboratory of Laser Materials and Devices, School of Physics and Electronic Engineering, Jiangsu Normal University, Xuzhou, China; 4 School of Optical and Electronic Information, Huazhong University of Science and Technology, 430074 Wuhan, China

**Keywords:** Optics and photonics, Lasers, LEDs and light sources

## Abstract

Soliton molecules (SMs) are stable bound states between solitons. SMs in fiber lasers are intensively investigated and embody analogies with matter molecules. Recent experimental studies on SMs formed by bright solitons, including soliton-pair, soliton-triplet or even soliton-quartet molecules, are intensive. However, study on soliton-binding states between bright and dark solitons is limited. In this work, the formation of such novel SMs in a fiber laser with near-zero group velocity dispersion (ZGVD) is reported. Physically, these SMs are formed because of the incoherent cross-phase modulation of light and constitute a new form of SMs that are conceptually analog to the multi-atom molecules in chemistry. Our research results could assist the understanding of the dynamics of large SM complexes. These findings may also motivate potential applications in large-capacity transmission and all-optical information storage.

## Abstract (web summary)

Three novel types of polyatomic soliton molecules are reported and they are conceptually analogues to the multi-atom molecules in chemistry.

## Graphical abstract

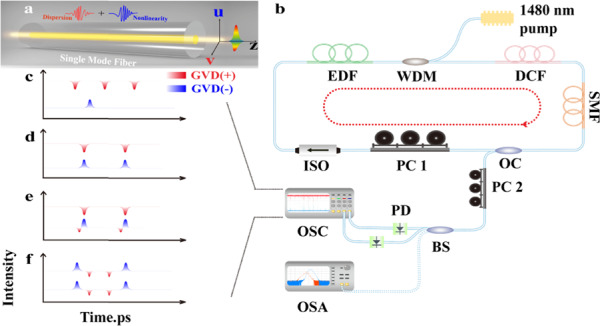

## Introduction

Solitons are localized wave packets that are ubiquitous in nature and have been studied in various physical fields, including Bose–Einstein condensates^1^, field theory^2^, and nonlinear optics^3^. Solitons exhibit particle-like behavior. Once formed, they can attract, repel, collide or annihilate upon interaction^4^. Via attractive and repulsive soliton interactions, multiple solitons can form a stably bounded state referred to as a soliton molecule^5–10^. In conservative systems, such as those described by the standard unperturbed nonlinear equation (NLSE), the interaction potential between solitons has no local minimum; thus, soliton molecules cannot be formed. A turning point occurred in the framework of coupled conservative systems. It is shown that the cross-phase modulation (XPM) between the orthogonally polarized components could drastically alter the scenarios of soliton interactions and give rise to a minimum in the interaction potential; therefore, bound solitons can be formed^11^. Specifically, the orthogonally polarized components propagating with slight differences in their propagation constants can be trapped and form a stable vector soliton state, known as a typical solution of the XPM-modified coupled nonlinear Schrödinger equations (CNLSEs)^12–22^.

Fiber lasers are widely used as an experimental testbed for investigations on soliton dynamics. SMs formed in fiber lasers are an intense research topic. Soliton-pair molecules that consist of two bound soliton pairs are the most studied multisoliton structures^23–25^. Beyond this simplest soliton molecule, solitons with more constituents, for example, soliton-triplet molecules^26^ and even soliton quartets composed of two soliton-pair molecules, have also been profoundly revealed in fiber lasers^27^. To our knowledge, nearly all these optical soliton molecules present closer analogies to the simple molecules of chemistry because bright solitons are their only constituent atoms. To date, studies on the compound type of soliton molecules, that is, the soliton-binding states of different types of solitons, e.g., dark and bright solitons, have rarely been conducted. In this work, we report various forms of such type of SMs in a fiber laser with near-zero group velocity dispersion (ZGVD). To highlight that the soliton molecules are made of different types of fundamental solitons, we named them “polyatomic” soliton molecules (PSMs). A summary of the observed PSMs in our fiber laser and their formation mechanisms are listed in Table 1. Numerical simulations are conducted on the coupled Ginzburg–Landau equations (CGLEs) corroborate these experimental findings. Physically, near ZGVD is a special dispersion regime where coexistence of dark solitons (DSs) and bright solitons (BSs), or even their vector forms, formed on either side of the ZGVD point, is permitted, which thus naturally increases the complexities of the system. The more solitons that participate in a SM, the higher the degrees of internal motion of the formed molecule. Analogous to the chemical counterparts formed by covalent bonds, in our work, the participation of multiple DSs and BSs in the molecules drastically enriches the binding schemes of the optical molecules. By showing a wide diversity of molecular bonds according to the type of soliton constituents and the coupling strength between them, which we could experimentally alter, we believe that the present work is of crucial importance in understanding the interaction dynamics of large soliton molecular complexes such as soliton supramolecules and soliton crystals. The results are of significance in both fundamental science and practical applications, for instance, these findings may motivate applications in multiple encoding, supercontinuum generation, and all-optical bit storage.

**Table 1:**
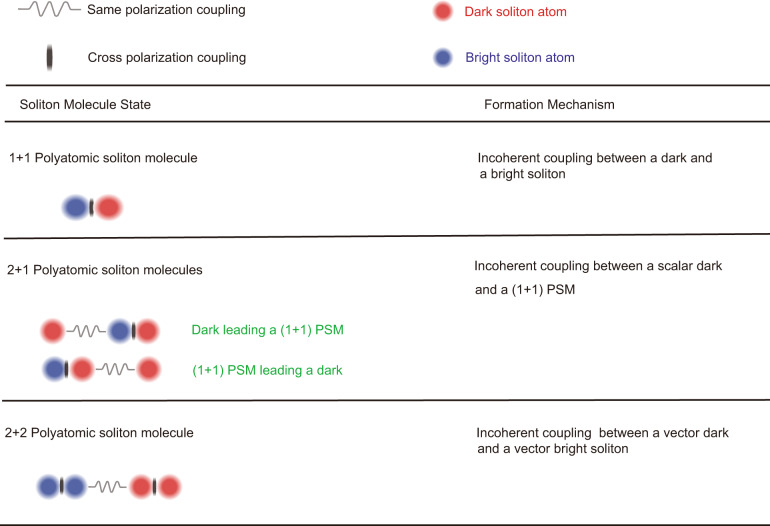
Summary on various observed soliton molecules and their formation mechanisms

A schematic illustration on the structure of each soliton molecule. Red-shaded circle: atomic representation for a dark soliton formed in the normal GVD regime; blue-shaded circle: atomic representation for a bright soliton formed in the anomalous GVD regime; spring and vertical stripe notations correspond to intramolecular bonds. Stripe notation: the intramolecular bond between orthogonally polarized solitons. Spring notation: the intramolecular bond between solitons with the same polarization and different wavelengths

## Results

### Experimental results

**Fig. 1:**
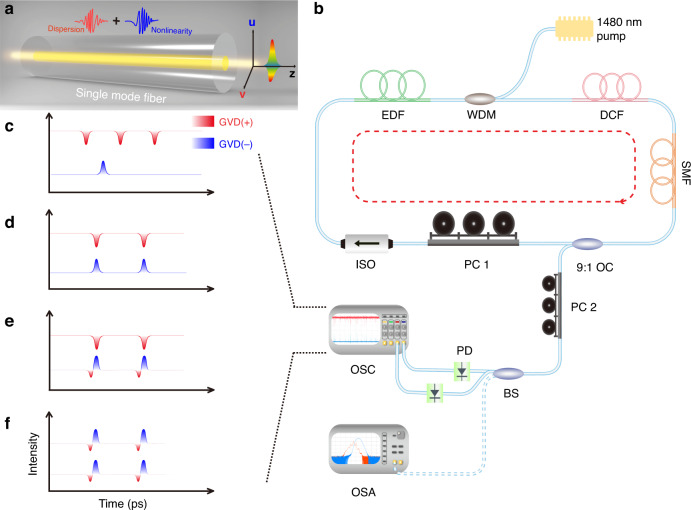
Schematic of fiber laser cavity and different soliton operation states. **a** Illustration of a birefringent single-mode fiber. **b** Configuration of the fiber ring laser. **c**–**f** Sketch of four typical soliton operation states observed in the experiments. Red color part: DSs formed in the net normal GVD; blue color part: BSs formed in the net anomalous GVD. **c** Unbound state of DSs and BSs. **d** State of (1 + 1) PSMs. **e** State of (2 + 1) PSMs. **f** State of (2 + 2) PSMs

We conducted the experimental studies in a quasi-vectorial cavity fiber laser as presented schematically in Fig. 1b. More information on the fiber cavity and the measuring systems are described in “Materials and methods” in Supplementary Materials.

#### Formation of (1 + 1) PSMs

**Fig. 2:**
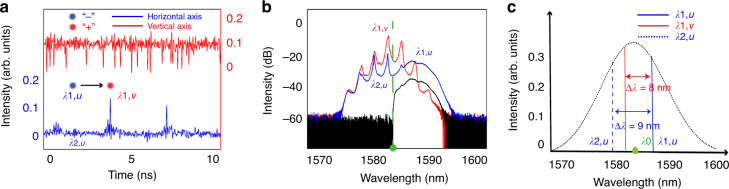
Simultaneous formation of orthogonally polarized DSs and BSs. **a** Polarization-resolved laser emissions. Blue solid line and red solid line: laser emission along two polarization axes. **b** The corresponding optical spectra of (**a**). **c** Schematic illustration on the soliton wavelengths. λ_0_: the position of ZGVD point, *λ*_1,*u*_: central wavelength of the BSs, *λ*_2,*u*_: central wavelength of dispersive waves, *λ*_1,*v*_: central wavelength of the DSs

A crucial difference of our fiber laser from the conventional mode-locked fiber lasers (MLFLs) is that no saturable absorber (SA) or any saturable-absorption mechanism exists in our laser cavity. Through taking the advantages of the dispersion- and birefringence-management techniques, experimentally we are able to control the averaged cavity dispersion close to the ZGVD point, where both the scalar DSs and BSs could coexist in a laser. A typical such laser operation state obtained at intracavity laser power ~18 mW is shown in Fig. 2. Figure 2a shows the laser emissions measured on oscilloscope. Obviously, scalar DSs and BSs are formed along orthogonal polarization axes of the cavity, respectively. The scalar BSs (DSs) exhibit typical characteristics of the scalar solitons obtained in the conventional SMF lasers^28,29^. In the current situation, because the wavelength separation between formed DSs and BSs, ∆*λ* = *λ*_1,*u*_ – *λ*_1,*v*_ = 8 nm, is quite large, there is a big group velocity mismatch between them, therefore, they are unbound and travel independently in the cavity. Figure 2b is the optical spectrum of Fig. 2a. The central wavelength of the DS locates at 1582 nm. We note that some periodic sidebands appearing on the spectrum, which are caused by the cavity detuning^30^. Similar spectral properties of dark soliton are also reported previously by Shao et al.^31^. In contrast, the spectrum of the BS exhibits some remarkable special features. Several research groups have investigated previously BS formation in single-mode fibers at or close to the ZGVD point^32,33^. It is shown that an optical pulse launched at or close to the ZGVD point of a fiber will lead to the emergence of BSs and their central frequency is shifted into the anomalous GVD. Owing to the mean pulse frequency conservation, a part of the pulse energy is radiated in the form of dispersive waves whose wavelengths are shifted into the normal GVD, forming a clear dip at the ZGVD point in the optical spectrum. Here, the spectrum of the BS well resembles this feature. Specifically, the spectrum comprises two parts: the left-side part, exhibiting several spectral spikes, belongs to the dispersive waves, while the right-side part resembles a Gaussian shape with the central wavelength located at 1590 nm; it belongs to the BSs. There is a clear dip appearing in the Fig. 2b, indicating the location of the ZGVD point. To verify that the BSs are generated in the anomalous GVD, we inserted a high pass filter at the laser output to filter out the components with a wavelength shorter than 1583 nm. The spectrum is displayed as the black trace in Fig. 2b. With this change, we found that BSs are still observed, except that the continuous-wave (CW) background on the BS side is reduced. Based on the formation requirements on scalar DSs and BSs, and the close resemblance of the BS spectra to those of solitons formed in fibers with close to ZGVD. We believe that the wavelength of ZGVD point of our laser cavity is around 1583 nm.

**Fig. 3:**
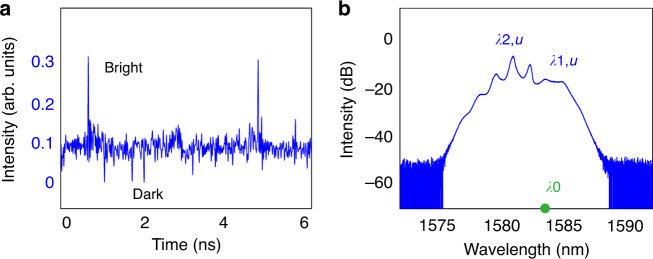
Scalar DSs and BSs simultaneously formed along the same polarization. **a** Coexistence of scalar BSs and DSs. **b** Optical spectrum of Fig. 3a. *λ*_0_: central wavelength of the ZGVD point, *λ*_1,u_: central wavelength of the BSs, *λ*_2,*u*_: central wavelength of the DSs

Scalar BSs and DSs can be simultaneously formed not only along the different polarization axes but also along the same polarization axis when the formation conditions are fulfilled. Previously, we have experimentally reported that scalar BSs and DSs could coexist along the same polarization in a fiber laser^34^. The key requirement for obtaining such a state is that the averaged cavity dispersion must be near the ZGVD point. In our current laser this state can also be easily obtained. Starting from the case as displayed in Fig. 2a, if we further increase the pump power, the CW level of the laser emission could also be increased. As the CW emission is in the normal GVD regime of the cavity, and the formation of small amplitude dark solitons is threshold-less^35^, when the CW intensity is increased to a sufficiently strong level, scalar DSs would automatically be formed as well, as presented in Fig. 3. Generally, when the central wavelength difference between the DSs and BSs is large, as shown in Fig. 3b, the attraction force generated by the incoherent cross-phase modulation between them cannot balance their group velocity mismatch, the solitons would be unbound. In this case, they will travel independently in the cavity. Otherwise, the BSs and DSs could be trapped as reported previously^34^.

Here, we will focus on the interaction between the BSs and DSs formed along the orthogonally polarization directions. It is well-known when light propagates inside a birefringent SMF, the cross-phase coupling between the orthogonally polarized light components may give rise to a two-component localized state, referred to as a vector soliton^36^. Theoretical studies have shown that relating to the sign of the dispersion, vector solitons made of various combinations of BS and DSs could be obtained. Among them, the vector solitons composed of a dark-bright (DB) soliton pair have attracted great attention. To date, vector DB solitons have been widely studied in both the spatial and temporal domains^16–22,37,38^. Table 2 summarizes the theoretical predictions on the vector DB solitons formed based on the CNLSEs.

**Table 2:**
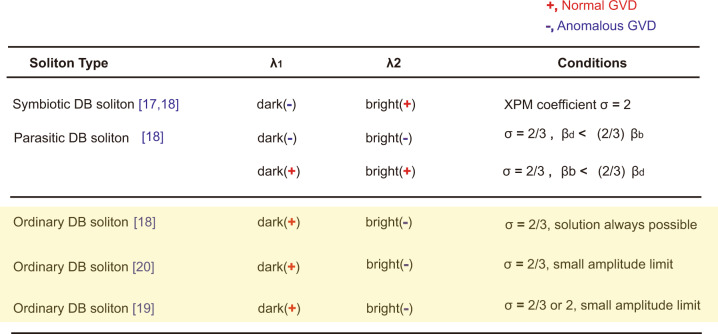
Theoretical predictions on vector dark-bright solitons and their formation conditions

Yellow shaded area: ordinary dark-bright solitons (ODBSs); “+” notation: soliton formed in normal dispersion regime; “−” notation: soliton formed in anomalous dispersion regime

The theoretically predicted vector DB solitons can be categorized into two groups: vector DB solitons whose components have no scalar counterparts and vector DB solitons whose formation can be treated as interaction between the existing solitons. The appearance of the former is purely a new feature of coupled nonlinear systems. Typical examples are the symbiotic and parasitic DB solitons. The concept of symbiotic DB solitons was first raised by Trillo et al. in 1988^17^. They showed that a DS propagating in the anomalous GVD could bind with a BS propagating in the anomalous GVD along the same polarization axis. In addition to symbiotic DB solitons, in 1990, Lisak et al. further predicted a group of parasitic DB soliton solutions based on the CNLSEs; It was shown that a BS (DS) can parasitize to a DS (BS) propagating in the normal (anomalous) GVD regime and form a stable bound state^18^. In parallel with studies on the symbiotic and parasitic DB solitons, Kivshar et al. theoretically predicted another group of DB soliton. They theoretically verified that within the small amplitude limit, the features of vector solitons are governed by an integrable model that can be solved using the inverse-scattering transformation^19–21^. As without coupling the DSs and BSs can already be formed individually, we named such a vector DB soliton an ordinary dark-bright soliton (ODBS). Notably, an ODBS can only be formed in the vicinity of the ZGVD point, while the formation of the symbiotic DB solitons or the parasitic DB solitons has no such constrain. Recently, both the parasitic DB solitons and the ODBS made of the same polarization dark-bright solitons were experimentally revealed in SMF lasers^37–39^. However, to our knowledge, no experimental observations on the ODBSs made of orthogonally polarized dark-bright solitons are reported.

**Fig. 4:**
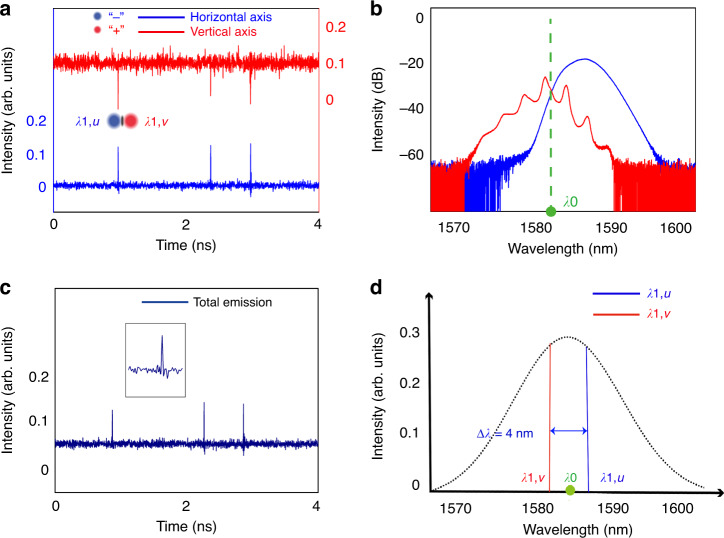
ODBSs ((1 + 1) PSMs) formed through incoherent cross-polarization coupling. **a** Polarization-resolved laser emissions. Blue solid line and red solid line: polarization-resolved laser emissions. **b** Corresponding optical spectrum of (**a**). **c** Total laser emission of (**a**). Inset: zoom-in of one BS pulse. **d** Schematic illustration of the soliton wavelengths. *λ*_0_: central wavelength of the ZGVD point, *λ*_1,*u*_: central wavelength of the BSs, *λ*_1,*v*_: central wavelength of the DSs

Although operating a fiber laser near the ZGVD point, simultaneous formation of scalar DSs and BSs of orthogonal polarizations could be relatively easily obtained, as shown in Fig. 2, owing to the smaller power threshold on the formation of solitons near the ZGVD point, the solitons formed have small energy, therefore, the attraction force between them is also weak. If the walk-off between them is relatively large, trapping between them would be difficult. In our fiber laser, the walk-off between solitons is related with the net cavity birefringence. Therefore, to achieve their trapping, one should possibly make the net cavity birefringence small. In our experimental studies, we use the wavelength difference between the orthogonally polarized CW laser emissions to reflect the strength of cavity birefringence. When we carefully varied the intracavity PC (IPC) paddles to reduce the central wavelength separation between the BSs and DSs. Under the pump strength of 21 dBm, when the wavelength difference ∆*λ* = *λ*_1,*v*_ – *λ*_1,*u*_ is reduced to about 4 nm, BSs and DSs are trapped as shown in Fig. 4a. Although the DSs could have different depths, once they are trapped by the BSs, they travel at the same speed with the BSs in the cavity. Figure 4b shows the corresponding optical spectra of Fig. 4a. The blue trace is the spectrum of BSs, its central wavelength locates at 1586 nm. The red trace is the optical spectrum of the DSs, whose central wavelength is located at 1582 nm. Obviously, the solitons are incoherently coupled. They are in fact a kind of ODBSs. We note that each of the ODBSs can also be interpreted as a kind of soliton molecule. Different from those of the soliton molecules reported so far in the literature that are formed through interaction between two BSs, the soliton molecules are made of a DS formed in the normal GVD regime and a BS formed in the anomalous GVD regime, as schematically illustrated in Fig. 4d. We denote the soliton molecules as a (1 + 1) polyatomic soliton molecule. It is interesting to note that once the trapping is occurred, then no free-running DSs are observed. Each DS is trapped by a BS, and they co-propagate in the cavity. To check if there is visible temporal offset between the trapped solitons, we also experimentally measured the total laser emission and compared the obtained pulse pattern with that of the BSs, as shown in Fig. 4c. As the BSs are much stronger than the DSs, in the total laser emission no signature of the DSs is visible, indicating that temporally the BSs and DSs are almost overlapped. Experimentally, the trapping state is quite sensitive to environmental perturbations. It could be easily destroyed. However, through carefully varying the IPC paddles, a similar soliton molecule state could always be re-established.

#### Formation of (2 + 1) PSMs

**Fig. 5:**
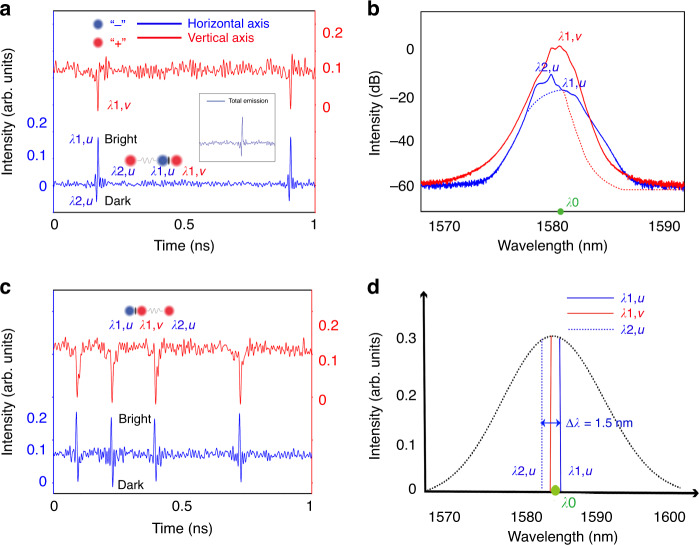
Two kinds of (2 + 1) PMSs experimentally observed. **a** A state of (2 + 1) PSMs where DSs are leading the ODBSs. Blue solid line and red solid lines: polarization-resolved laser emissions. Inset: total laser emission of each (2 + 1) PSM. **b** Polarization-resolved optical spectra of (**a**); blue (red) dashed-line fitted part: spectrum for the BSs (DSs) formed at the wavelength *λ*_1,*u*_ (*λ*_2,*u*_), *λ*_0_: central wavelength of the ZGVD point. **c** Another state of the (2 + 1) PSMs where the ODBSs are leading the DSs. **d** Schematic of the wavelength relationship of the DSs and BSs, *λ*_0_: the position of ZGVD point, *λ*_1,*u*_: central wavelength of the BSs formed along the horizontal polarization axis, *λ*_2,*u*_: central wavelength of the DSs formed along the horizontal axis, *λ*_1,*v*_: central wavelength of the DSs formed along the vertical polarization axis

Starting from a (1 + 1) PSM state as presented in Fig. 4a, if further increase the pump power, e.g., to 23 dBm (intracavity laser power: ~20 mW) in our experiment, the CW along the horizontal polarization axis could also increase to a sufficient power level where small amplitude scalar DSs automatically appear along one polarization axis. A similar formation mechanism of DS is described above for the result shown in Fig. 3. Normally, the DSs are formed randomly, and they move with different velocities from the (1 + 1) PSMs. However, through varying the IPC paddles so that the central wavelength separation between the scalar DSs and BSs becomes very small, e.g., ∆*λ* = *λ*_1,*u*_ – *λ*_2,*u*_ = 1.5 nm, the DSs could be trapped by the (1 + 1) PSMs, forming a new PSM state, as shown in Fig. 5a. We denote such a soliton trapping state as a (2 + 1) PSM. Figure 5b shows optical spectra of the state. The spectrum along the horizontal axis is obviously composed of two parts: the spectrum fitted with blue dashed-line-fitted belongs to the BSs of the ODBS, whose central wavelength locates at 1583.5 nm, the red dashed-line-fitted spectrum belongs to the scalar DSs, whose central wavelength locates at 1582 nm. Notably, the wavelength of the DSs is located in between the above two wavelengths. To highlight the wavelength relationships of the trapped solitons, we also draw their positions schematically with respect to the ZGVD point in Fig. 5d. Such a wavelength relationship ensures a strong incoherent coupling among them. Experimentally, we observed two types of (2 + 1) PSM profiles: in one profile, the dark soliton is leading the ODBS, as shown in Fig. 5a, while in the other profile, the ODBS is leading the DS, as presented in Fig. 5c. Which of the (2 + 1) PSM profiles would be formed is determined by the specific experimental conditions, which are discussed in Supplementary Section 6. Once a (2 + 1) PSM is formed, the BSs and DSs within the PSMs no longer undergo any noticeable change. The three solitons move stably as an entity. Worth of noting that different from the trapping between BSs and DSs with orthogonal polarizations, there is clearly a visible temporal offset between the trapped DSs and DSs, as shown in the inset of Fig. 5a, suggesting that their detailed coupling features are different.

We experimentally measured the autocorrelation trace of the BSs; If assuming it has a sech^2^ pulse shape, the pulse width is about 2 ps. The pulse width of the DSs could also be in the level of picoseconds. As shown in Fig. 5a, c, the separations between the (2 + 1) PSMs are generally on the order of hundreds of soliton pulse widths. Hence, they are expected to form weaker bonds characterized by long-range interactions. Unlike short-range interactions, long-range interactions are phase independent and could be caused by different mechanisms, including acoustic effects^40^, laser gain depletion and recovery^41^, dispersive waves^42,43^, unstable continuous waves^44^, Casimir-like effects^45^, thermal effects^46^, optomechanical effects^47^, and more recently, intracavity reflexes^48^. No wonder that partial or all these effects could exist and perturb the soliton interactions. However, in a cavity fiber laser like our one, the solitons are robust attracting states of the nonlinear resonator, tied to the phase and carrier frequency of a coherent cavity field. They can hardly shift their carrier frequencies. Therefore, the acoustic effect on the solitons is much weaker than that in single-pass systems^49^. On the other hand, as the temporal separations between the solitons are normally much shorter than the recovery time of the erbium-doped fibers (EDF) which has a typical value of ms^50^. The laser gain and depletion-mediated long-range interactions in an EDF laser are also ultraweak. To date, several research groups have both numerically and experimentally evidenced that in a fiber laser, the long-range interactions between solitons are mainly caused by the dispersive waves (DWs) and unstable CW components^51^. In our experiment in order to obtain PSMs, the strength of CW must be sufficiently strong so that DSs could be formed. It was noticed experimentally when the strength of CW and/or the DWs is strong, an initial equally spaced BS pulse train could become unequally spaced, as presented in Supplementary Fig. S3c in Supplementary Section 3 and Supplementary Visualization 3, suggesting the long-range soliton interaction also exist in our laser system under the laser operation situation. Relating to the specific laser operation conditions and the environmental perturbations, experimentally other forms of soliton dynamics that could be unambiguously traced back to the long-range soliton interaction are also observed, as shown in Supplementary Visualization 4. Like the multiple BS operation, due to the long-range intermolecular interactions^51^, occasionally two or multiple (2 + 1) PSMs could also move sufficiently close to each other, forming complicated (2 + 1) PSM dynamics, such as a restless kind of relative molecular movement^52^, or different forms of (2 + 1) PSM bunches, etc. However, compared to the intramolecular binding strength, the intermolecular coupling is much weaker. Therefore, the formed PSM bunches can be experimentally easily altered through varying the PC paddles or increasing the intracavity power.

#### Formation of (2 + 2) PSMs

**Fig. 6:**
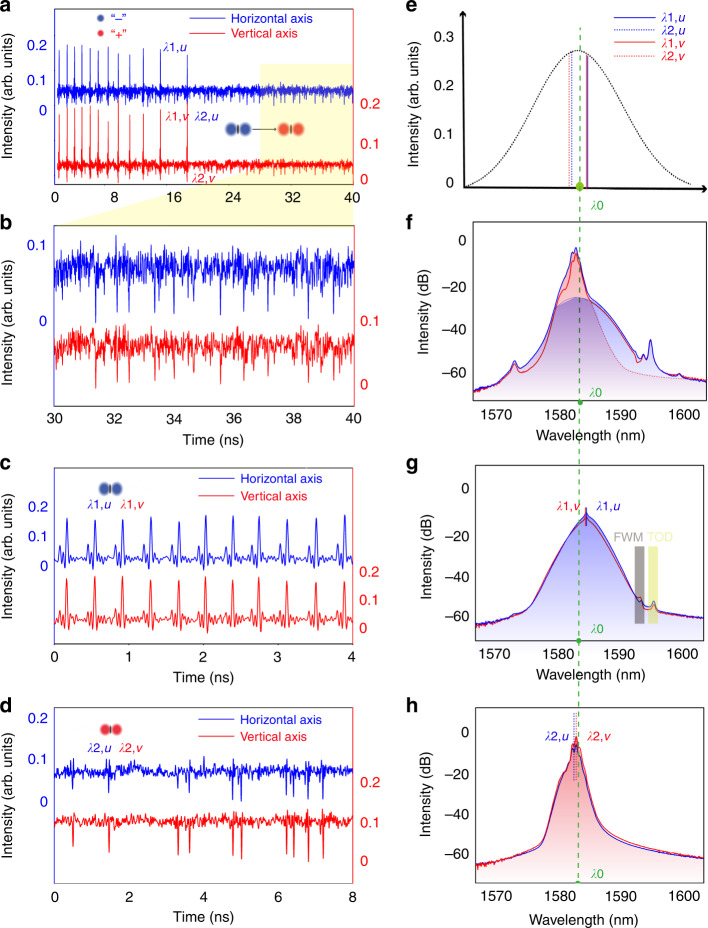
Coexistence of vector BSs and DSs. **a** State of coexistence of vector BSs and DSs. **b** Zoom-in of the vector DSs in the time window from 30 to 40 ns as shown in (**a**). **c** State of vector BSs. **d** State of vector DSs. **e** Schematic of the laser gain curve. Central wavelengths of the BSs and DSs across the ZGVD point, *λ*_0_: the position of ZGVD point, *λ*_1,*u*_: central wavelength of BSs formed along the horizontal polarization axis, *λ*_2,*u*_: central wavelength of the DSs formed along the horizontal axis, *λ*_1,*v*_: central wavelength of the BSs formed along vertical polarization axis, *λ*_2,*v*_: central wavelength of DSs formed along vertical polarization axis. **f**–**h** Optical spectra of (**a**, and **c**, **d**). **f** Red color part: fitted spectrum of the vector DSs according to (**h**), blue color part: fitted spectrum of the vector BSs according to (**g**). **g** Spectrum for BSs. **h** Spectrum for DSs

Experimentally, both the lasing intensity and net cavity birefringence along the orthogonally polarized axes can be altered by varying the orientations of the IPC paddles and the pump power. Under a suitable high pump power, e.g., 30 dBm (intracavity laser power: ~40 mW) in our experiment, if the net linear cavity birefringence is tuned sufficiently weak, as judged by that the wavelength difference between the two orthogonally polarized CW laser emissions has a very small value, a state with the coexistence of incoherently coupled vector DSs and coherently coupled vector BSs as shown in Fig. 6a could be obtained in our fiber laser. A video of the state is also shown in Supplementary Visualization 1. As can be seen in Fig. 6a, dozen pairs of vector BSs are formed in the anomalous GVD regime of the cavity. Due to indirect soliton interactions, the vector BSs form a soliton bunch. If one triggers the oscilloscope traces with the BSs, then all the BSs are frozen, while the DSs still move in the cavity. We also numerically verified this coexistence state of DSs and BSs in Supplementary Section 5. Figure 6b shows a zoom-in of the time window from 30 to 40 ns in Fig. 6a. Except for the random background noise, the dark pulses are obviously well coupled to each other, suggesting that the observed intensity dips are vector DSs rather than noise or any other artifices generated by the detection system.

Under the state shown in Fig. 6a, if the orientation of the PC paddles is slightly tuned, another state as shown in Fig. 6c can be obtained, where only the vector BSs exist, or that shown in Fig. 6d, where only the vector DSs exist. Figure 6g, h shows the corresponding optical spectra of Fig. 6c, d. Both the four-wave mixing (FWM) sidebands and a weak unsymmetrical Kelly sideband are visible in Fig. 6g, suggesting that the vector BSs are phase-locked BSs, and the asymmetries in the Kelly sidebands is generated by the third-order dispersion (TOD)^53^. The central wavelength of the vector BSs is near 1585 nm, while that of the DS is near 1582 nm. Comparing the results shown in Fig. 6g, h to those in Fig. 6f, obviously, the optical spectrum in Fig. 6f consists of two parts, specifically, the blue-shaded part for vector BSs, and the red-shaded part for the DSs. These results further confirmed that the ZGVD wavelength of our fiber laser is near 1583 nm, and the vector DSs and BSs are formed on either side of the ZGVD point. We stress that this state is only possible in a fiber laser operating near the ZGVD point with a sufficiently broad effective gain bandwidth.

**Fig. 7:**
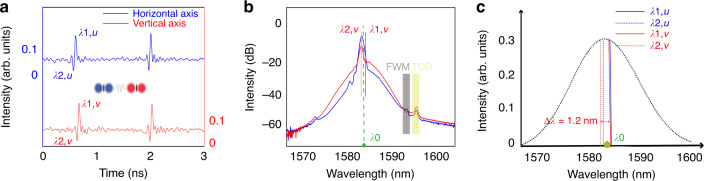
Formation of (2 + 2) PSMs. **a** A state of (2 + 2) PSMs. Blue solid line and red solid line: polarization-resolved laser emissions. **b** Optical spectra of (**a**). **c** Schematic illustration of the central wavelengths of the vector BSs and DSs with respect of the ZGVD point, *λ*_0_: the position of ZGVD point, *λ*_1,*u*_: central wavelength of BSs formed along the horizontal polarization axis, *λ*_2,*u*_: central wavelength of the DSs formed along the horizontal polarization axis, *λ*_1,*v*_: central wavelength of the BSs formed along vertical polarization axis, *λ*_2,*v*_: central wavelength of the DSs formed along vertical polarization axis. Each (2 + 2) PSM is made up of a pair of coherently coupled BSs and incoherently coupled DSs

We point out that each pair of the vector BSs and DSs can also be interpreted as a (1 + 1) soliton molecule. Although in the state shown in Fig. 6a the net birefringence is already very weak, the vector BSs and DSs move independently in the cavity, indicating that the wavelength difference between the formed BSs and DSs ∆*λ* = *λ*_1,*u*_ – *λ*_2,*v*_ = 3 nm, is still too large so that the walk-off between the vector solitons could not be balanced by their attractive interaction force. Therefore, to achieve trapping of them, we experimentally further reduced the wavelength separation ∆*λ* = *λ*_1,*u*_ – *λ*_2,*v*_ to ~1.2 nm, a state as presented in Fig. 7a is observed, where the vector DSs are trapped by the vector BSs. We denote such a soliton trapping state as a (2 + 2) PSM. Figure 7b shows the polarization-resolved optical spectra of Fig. 7a. To highlight the wavelength relationships of the solitons, we draw again schematically their positions with respect to the ZGVD point in effective gain curve in Fig. 7c.

Again, it is noticed in our experiment that once the (2 + 2) PSMs are formed, no free-moving vector DSs are visible in the cavity. In such a laser operation state, each vector DS is trapped by a vector BS through incoherent coupling, and the trapped vector solitons move as an entity in the cavity. Especially, the temporal offset between the trapped DS and BS no longer changes. A typical time evolution of the trapped vector soliton is extracted in Supplementary Fig. S2 in Supplementary Section 2. Slightly altering the laser operation parameters would not destroy the state, indicating that the coupling forces among the solitons are very strong. In optical communication systems, working near the ZGVD point is desirable because of the low power threshold for creating solitons. In addition to this well-known merit, near ZGVD is a special regime where the simultaneous formation of DSs and BSs solitons is possible, as shown above, permitting the formation of stable compound soliton molecules made up of both DSs and BSs. Such soliton molecules would have drastically increased degrees of freedom, holding promise for applications in multiple encoding. Specifically, if the soliton molecules were used as information carriers, then the capacity of telecommunication systems could be significantly increased by providing extra degrees of coding.

### Numerical simulations

**Fig. 8:**
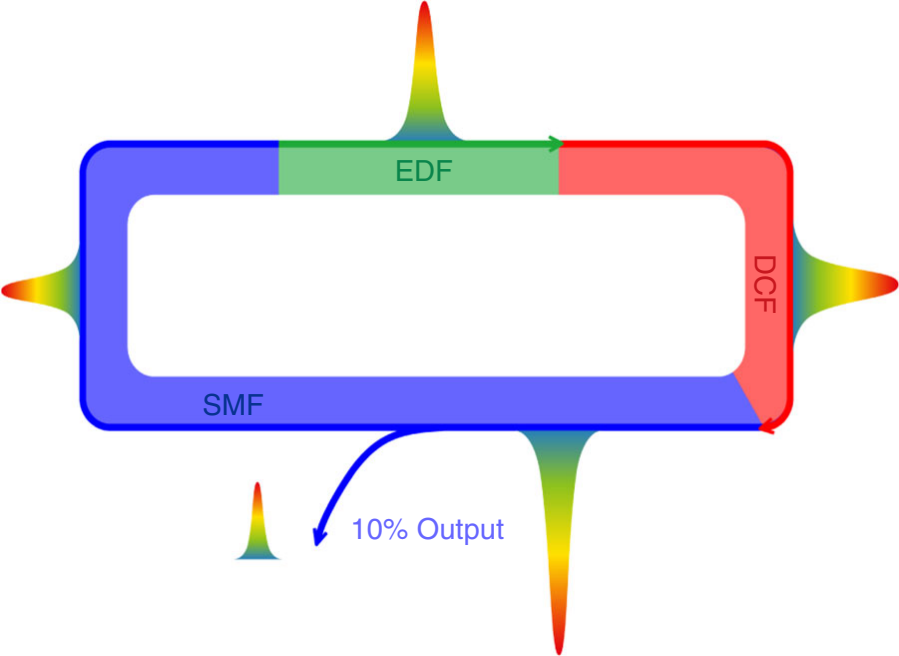
Schematic illustration of the numerical simulation model. EDF erbium-doped fiber, SMF single-mode fiber, DCF dispersion-compensation fiber

To provide better insight into the experimental observations, we also numerically simulated various laser operation states of our fiber laser. A schematic of the numerical simulation model is presented in Fig. 8. The simulation model is based on the following CGLEs:

1 $$\begin{array}{c}\frac{{\partial u}}{{\partial z}} = + \delta \frac{{\partial u}}{{\partial t}} - \frac{{i\beta _{2u}}}{2}\frac{{\partial ^2u}}{{\partial t^2}} + \frac{{\beta _{3u}}}{6}\frac{{\partial ^3u}}{{\partial t^3}}\\ + i\gamma (\left| u \right|^2 + \frac{2}{3}\left| v \right|^2)u + \frac{g}{2}u + \frac{g}{{2\Omega _g^2}}\frac{{\partial ^2u}}{{\partial t^2}}\\ \frac{{\partial v}}{{\partial z}} = - \delta \frac{{\partial v}}{{\partial t}} - \frac{{i\beta _{2v}}}{2}\frac{{\partial ^2v}}{{\partial t^2}} + \frac{{\beta _{3v}}}{6}\frac{{\partial ^3v}}{{\partial t^3}}\\ + i\gamma (\left| v \right|^2 + \frac{2}{3}\left| u \right|^2)v + \frac{g}{2}v + \frac{g}{{2\Omega _g^2}}\frac{{\partial ^2v}}{{\partial t^2}}\end{array}$$

**Fig. 9:**
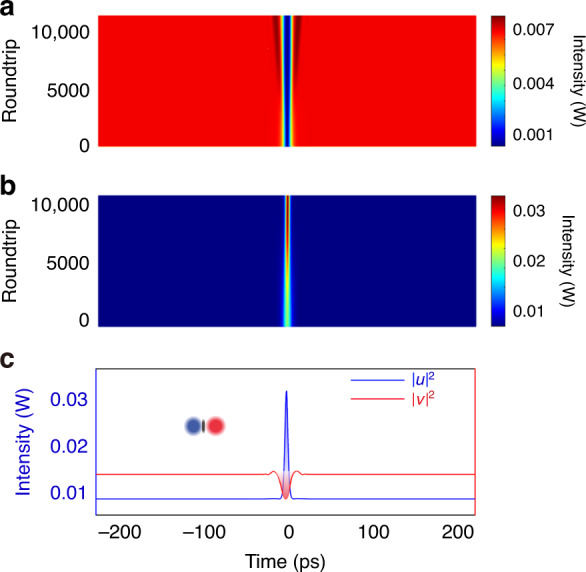
A typical ODBS state numerically simulated. More details can be found in Section 4 in Supplementary Material. Parameters used in the simulation: $A = 0.1;B = 1;\;\beta _{2u,ave} = 0.01\;\mathrm{ps}^2\;\mathrm{km}^{ - 1};\;\beta _{2v,ave} = - 0.01\;\mathrm{ps}^2\;\mathrm{km}^{ - 1};$ $\beta _{3u,ave} = \beta _{3v,ave} = 0.001\;\mathrm{ps}^3\;\mathrm{km}^{ - 1};$ $g = g_0/(1 + {\int} {\left( {\left| u \right|^2 + \left| v \right|^2} \right)} \mathrm{d}t/E_s)$; $g_0 = 100\;\mathrm{km}^{ - 1};$ $E_s = 1\;\mathrm{pJ};\;\gamma = 3\;\mathrm{W}^{ - 1}\;\mathrm{km}^{ - 1};\;\delta = 0.0005\;\mathrm{ps}\;\mathrm{km}^{ - 1}$. **a** Evolution of DS formed in the normal cavity GVD. **b** Evolution of BS formed in the anomalous cavity GVD. **c** Red solid line and blue solid line: DS and BS observed at the last cavity round trip of 10,000

**Fig. 10:**
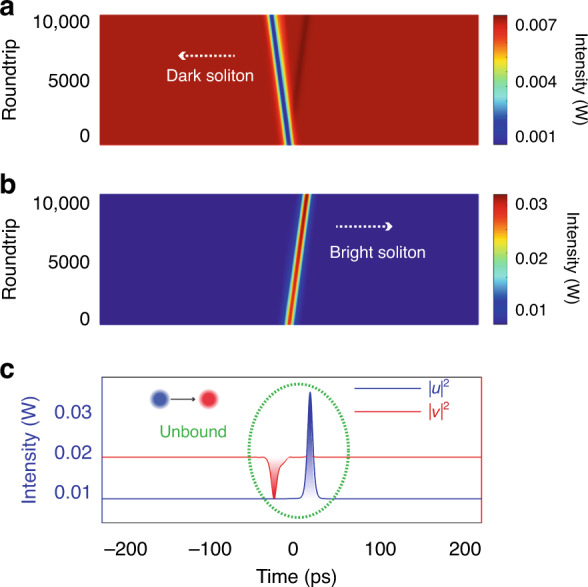
A typical independently propagating state of DSs and BSs numerically obtained. The same simulation parameters as in Fig. 9 are used except for a larger group velocity mismatch of δ = 0.05 ps km^−1^. **a** Evolution of DS formed in the normal cavity GVD. **b** Evolution of BS formed in the anomalous cavity GVD. **c** Red solid line and blue solid line: DS and BS observed at the last cavity round trip of 10,000

To faithfully simulate the laser operation, the laser cavity output, laser gain saturation, and the cavity boundary condition are also accounted. More details on the simulation model and the used numerical techniques are discussed in “Materials and methods” and Supplementary Section 4. We first simulated the formation of the (1 + 1) polyatomic soliton molecule. We start the simulation with a weak bright pulse in the form of sech (*A*∙*t*) and a dark pulse in the form of tanh (*A*∙*t*). Here, A is a constant account for the pulse width. Numerically, we deliberately vary the GVD coefficients of the SMF for the DSs and BSs for the case of incoherent coupling. In particular, we let bright pulse and dark pulse propagate in different GVD regimes. When the walk-off between the bright and dark pulses is small, a stable (1 + 1) PSM is observed in the laser, as shown in Fig. 9. The initial weak pulses quickly evolve into a pair of DB solitons, and they co-propagate in the cavity as an entity. Notably, the DS formed near the ZGVD point is slightly broader than the BS. This is different from the symbiotic DB solitons formed in slightly larger GVD regimes. The results agree well with the results obtained in Fig. 4a. We also considered the case of the large walk-off between the dark and bright pulses. The simulation results are displayed in Fig. 10, where, with the large group velocity mismatch, i.e., *δ* = 0.05 ps km^−1^, the DSs and BSs are not bound to each other. They are independently formed, which well matches the experimental results shown in Fig. 2a.

## Discussion

Different from the conventional MLFLs where, owing to the existence of a saturable absorber (SA) or a saturable-absorption-like mechanism in the cavity, only BSs can be observed, we have shown experimentally that without a SA in the cavity, fundamental DSs can be easily generated in a fiber laser. Specifically, through operating the fiber laser close to the ZGVD point, simultaneous formation of BSs and DSs, or even vector BSs and DSs, could occur. Not only the BSs could attract each other to form a simple soliton molecule, our experimental results have further shown that the formed BSs and DSs could also interact attractively to form a bound state of them. Worth of noting that all the experimental results observed could also be well governed by the scalar and/or coupled NLSEs, suggesting that a fiber laser is an ideal platform for conducting experimental studies on various NLSE types of solitons.

As particle-like wave packets, the interaction between different types of solitons has been a topic of considerable interest. Our experimental results could also shed light on the understanding of the long- and short-range interactions among solitons, which could open a way to create large optical molecules, e. g. solitonic supramolecules^54,55^. The elementary diversity of supramolecular structures can be greatly enriched by incorporating miscellaneous fundamental building blocks into the molecule, for example, different types of soliton atoms. The development of compound soliton molecules would largely increase the degrees of freedom of the molecular complexes, on the other hand, finding the complex dynamics that resemble the collective excitations of chemical compound molecular structures is of great importance. The concept of compound soliton molecules is also of practical significance because it could largely enrich the control schemes for optical soliton molecules, for example, by providing the possibility to control both soliton types and numbers instead of only soliton numbers. Although the SMF laser employed here is a one-dimensional optical platform, by introducing spatiotemporal nonlinearities into the laser, e.g., by using multimode fibers or photonic crystal fibers, higher-dimensional spatiotemporal solitons could be formed^56^. The development of spatiotemporal solitons could further advance the understanding of soliton formation and soliton dynamics of complex three-dimensional systems (for instance, proteins and lipids in biochemistry) where superstructural soliton molecules could be formed.

## Materials and methods

### Experimental setup and measurement system

The experimental setup is sketched in Fig. 1b. The fiber ring cavity consists of different segments of fibers, EDF, DCF, and stand SMF. By carefully selecting the length of each segment of the fiber, the net cavity dispersion is tuned to the vicinity of the ZGVD point. More details on the experimental parameters and measurement systems are listed in Supplementary Section 1.

## Supplementary information

Supplementary Information for Novel optical soliton molecules formed in a fiber laser with near-zero net cavity dispersion

Visualization S1. Experimental demonstration on the coexistence of vector bright and vector dark solitons in a fiber laser

Visualization S2. Experimental demonstration on the equally spaced bright solitons in a fiber laser

Visualization S3. Experimental demonstration on the unequally spaced bright solitons in a fiber laser

Visualization S4. Experimental demonstration on the bright soliton bunches in a fiber laser

## Data Availability

The data that support the simulations within this paper and other findings of this study are available from the corresponding authors upon reasonable request.
