# Supplementary material for: Novel optical soliton molecules formed in a fiber laser with near-zero net cavity dispersion
Source: Light Sci Appl. 2023 Feb 7;12:38. doi: 10.1038/s41377-023-01074-w (PMC9902550; doi:10.1038/s41377-023-01074-w)
Supplement: Supplementary file 1 — Supplementary Information for Novel optical soliton molecules formed in a fiber laser with near-zero net cavity dispersion [file 41377_2023_1074_MOESM1_ESM.pdf]

Supplementary Information for

Novel optical soliton molecules formed in a fiber laser with near-zero net cavity dispersion

Xiao Hu,[1,2] Jun Guo,[3] Jun Wang,[3] Jie Ma,[3] Luming Zhao,[4] Seongwoo Yoo,[2] and Dingyuan Tang[1,]*

[1]Julong College, Shenzhen Technology University, Shenzhen 518118, China

[2]School of Electrical and Electronic Engineering, Nanyang Technological University, Singapore 639798, Singapore

[3]Jiangsu Key Laboratory of Laser Materials and Devices, School of Physics and Electronic Engineering, Jiangsu Normal University, Xuzhou, China

[4]School of Optical and Electronic Information, Huazhong University of Science and Technology, Wuhan 430074, China

**Corresponding author. E-mail: tangdingyuan@sztu.edu.cn*

This Supplementary Information consists of the following sections:

--Section S1. A schematic of the fiber ring cavity.

--Section S2. Stability of the trapped dark-bright vector solitons.

--Section S3. Investigation on indirect soliton interactions.

--Section S4. Simulation model and numerical technique used.

--Section S5. Simulation results on coexistence of dark and bright solitons.

--Section S6. Simulation results on soliton trapping of dark-bright solitons.

--Supplementary Videos

--Supplementary References

Section 1: A schematic of the fiber ring cavity.

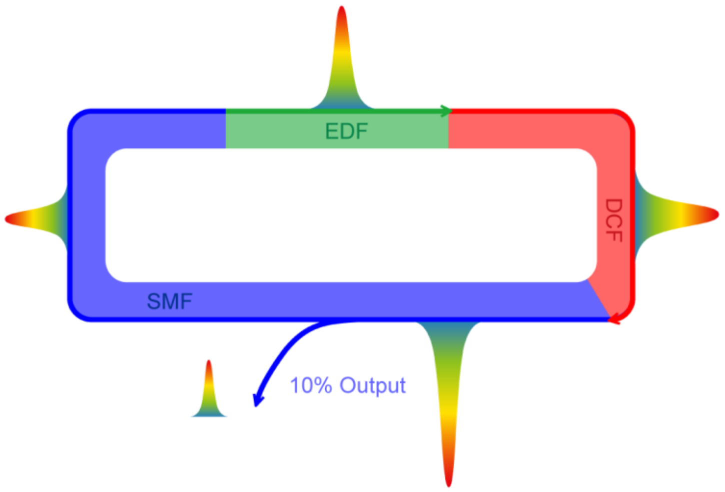

Fig. S1 Schematic of the fiber ring cavity used for the numerical simulations. Blue solid line: Single mode fiber; Green solid line: Erbium-doped fiber; Red solid line: Dispersion compensation fiber. erbium-doped fiber (OFS-EDF80 with GVD coefficient of $\beta_2 = 63.4\, ps^2\ km^{-1}$, dispersion shifted fiber (DCF with GVD coefficient of $\beta_2 = 5.1\, ps^2\ km^{-1}$) and stand single mode fiber (SMF-28 with GVD coefficient of $\beta_2 = -23.8\, ps^2\ km^{-1}$).

Experimentally, to separately observe the two orthogonal polarization components of the light field, the laser output is first sent to a fiber pigtailed polarization beam splitter and then monitored with a high-speed detection system consisting of two 40GHz photodetectors (Newport, Model 1014) and a 33GHz bandwidth real-time oscilloscope (Agilent Technologies, DSA-93204 A). An extra polarization controller (PC2) is inserted between the laser output and the beam splitter to balance the linear polarization change induced by the lead fibers. Finally, an optical spectrum analyzer (Yokogawa, AQ6375) is used to monitor the optical spectrum of the laser emission.

To make the simulation results directly comparable with the experimental observations, our simulations were conducted based on the real experimental fiber laser configuration as shown in Fig. S1. We adopt a pulse tracing technique to simulate the laser operation [1.] Briefly, when a light pulse circulates inside the cavity, the local fiber group velocity dispersion and birefringence varies with the fiber used. At different positions of the cavity, the pulse may have slightly different pulse shapes and energies. We note that as our cavity length is much shorter

than the pulse dispersion and nonlinearity length, the dispersion-managed features are not dominant. Numerically, we have output the light pulse at different positions of the ring cavity and verified that the pulse properties are mainly determined by the average cavity parameters rather than that of the single segment of the fiber ring laser [2].

Section 2: Stability of the trapped dark-bright vector solitons.

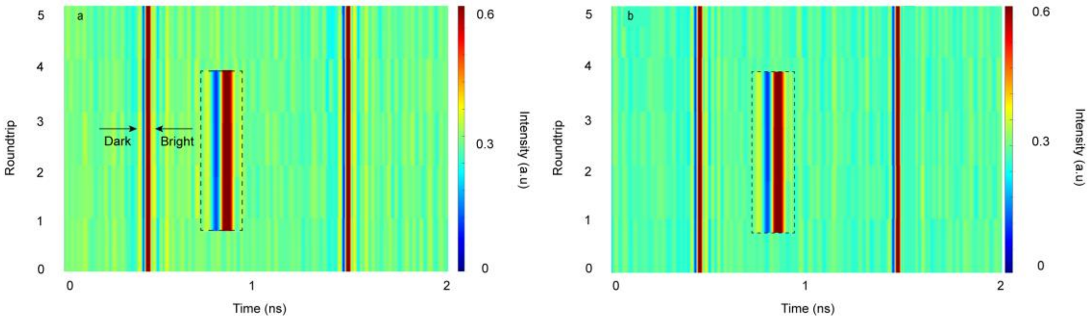

Fig. S2 Experimentally measured evolution of trapped dark-bright vector solitons with the cavity roundtrips (only along one polarization axis is displayed). Red vertical stripes: bright solitons. Blue vertical stripes in front of the red vertical stripe: dark solitons. Insets in a and b: Zoom in of the trapped dark-bright solitons. a. A typical trapping state measured at t=0 s. **b.** the same state measured at t=60 s.

Experimentally, by operating the fiber laser at very close to the ZGVD point, due to the sufficiently broad effective gain bandwidth of the erbium-doped fiber laser, the state of coexistence of vector dark and vector bright solitons is possible. Specifically, the vector dark and bright solitons are formed at two sides of the ZGVD point. Under large group velocity mismatch, they travel independently in the cavity as shown in Fig. 6 in the article. Starting from the state as shown in Fig. 6, when the wavelength difference between the dark and bright vector solitons becomes small so that their group velocity mismatch also becomes small, eventually the vector dark solitons will be trapped by the vector bright solitons and they will move as an entity inside the cavity, as shown in Fig. S2a. Fig. S2b shows the same vector dark-bright soliton pairs extracted at t = 60 s. It shows that this trapping state is very stable. From the experimental result it is also to see, if once formed, the temporal offset between the dark and bright solitons nearly remains unchanged, suggesting a stable bond between them.

Section 3: Investigation on indirect soliton interactions.

In the article, we have focused our studies on the short-range soliton interactions between dark and bright solitons. In particular, we showed that three types of "polyatomic soliton molecules" could be formed as a result of direct soliton interactions between the dark and bright solitons, either along orthogonally polarized axes (XPM coefficient σ=2/3) or along the same polarization axis but with different central wavelengths (XPM coefficient σ=2). In parallel with the short-range soliton interactions, in the case of moderate soliton separations, the solitons could also form weaker bonds caused by long-range interactions [3-18]. In the following section, we will provide some experimental evidence to show the existence of the long-range soliton interactions under certain laser operation conditions and their influences on the observed soliton dynamics.

We start from a typical bright soliton emission state as shown in Fig. S3a. Experimentally, by operating the fiber laser in the net anomalous cavity dispersion regime, bright solitons could be easily produced as shown in Fig. S3a. Fig. S3b is the corresponding optical spectrum of Fig. S3a. Without obvious existence of unstable CW or strong dispersive waves (DWs) in the cavity, all the bright solitons are equally spaced and show the soliton quantization feature. If one triggers the oscilloscope with one bright soliton, all the bright solitons are fixed in the oscilloscope trace as shown in Supplementary visualization 2. However, when the pump intensity is further increased, either the CW could become unstable or strong DWs could appear as shown in Fig. S3d. Consequently, the unstable CW or dispersive waves mediated long range soliton interaction starts to play a role, which causes relative soliton movement in the cavity as shown in Supplementary visualization 3. Eventually the bright solitons become unequally distributed in the cavity as presented in Fig. S3c. Depending on the specific experimental conditions and/or environmental perturbations, soliton bunches could also be frequently formed under the dispersive waves mediated long range soliton interaction. Such a soliton bunch is also be regarded as a kind of soliton molecule in the literature. However, compared to the soliton molecules formed through the direct soliton interactions, these soliton

molecules are only weakly stable. They could be easily destroyed by changing the experimental conditions, e.g., pump intensity or intracavity PC orientation. In addition of the weakly stable soliton bunches, another form of soliton bunches as shown in Supplementary visualization 4 can also be formed, where the solitons are also bound by the DWs mediated long-range soliton interaction, but the relative soliton positions constantly vary.

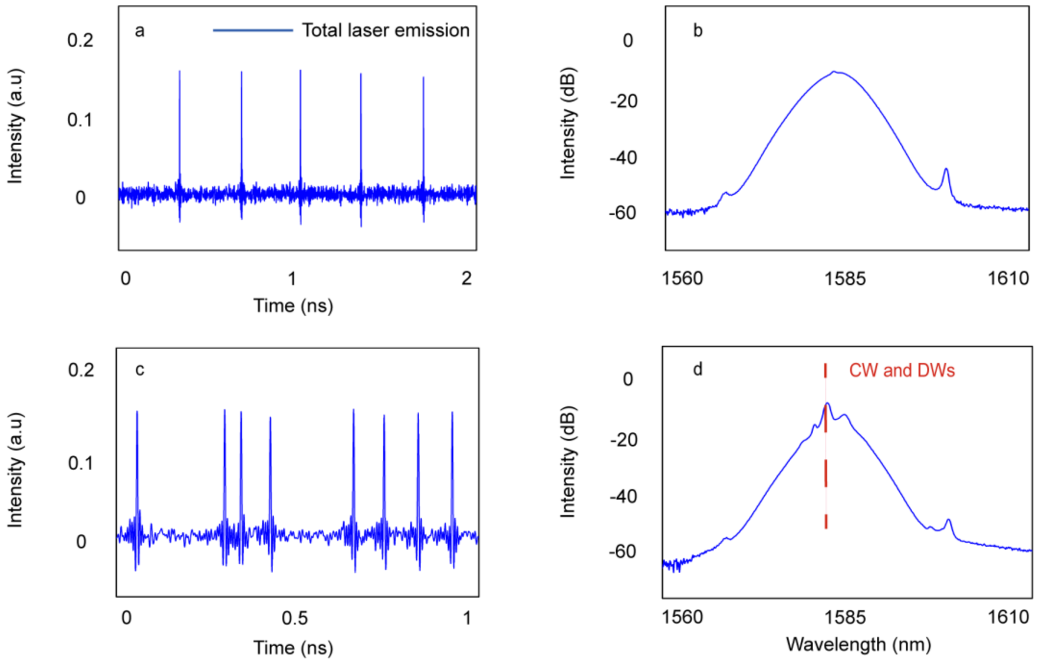

Fig. S3 Experimentally measured bright soliton emission state. a. Equally spaced bright solitons with absence of CW or DWs. **b**. Optical spectrum for a. **c**. Randomly distributed bright solitons in the presence of unstable CW or strong DWs. **d**. Optical spectrum for c.

Starting from a state as shown in Fig. S3c, if one further increases the intracavity power or varying the intracavity PC paddles until the strength of the CW becomes strong, dark solitons could be further formed in the normal dispersion regime and coexist with the bright soliton bunch as shown in Fig. 6 and Supplementary visualization 1 of the article. Experimentally, we have showed that under strong coupling between dark and bright solitons, a type of 2+2 PSMs could even be formed as presented in Fig. 7. It is to note that the existence of unstable CW or strong DWs will unavoidably influence the dark-bright soliton dynamics through the indirect soliton interactions (long-range interactions). Therefore, similar to the formation of the state

shown in Fig. S3c, random irregularly distributed dark-bright soliton molecules as shown in Fig. S4 could be formed.

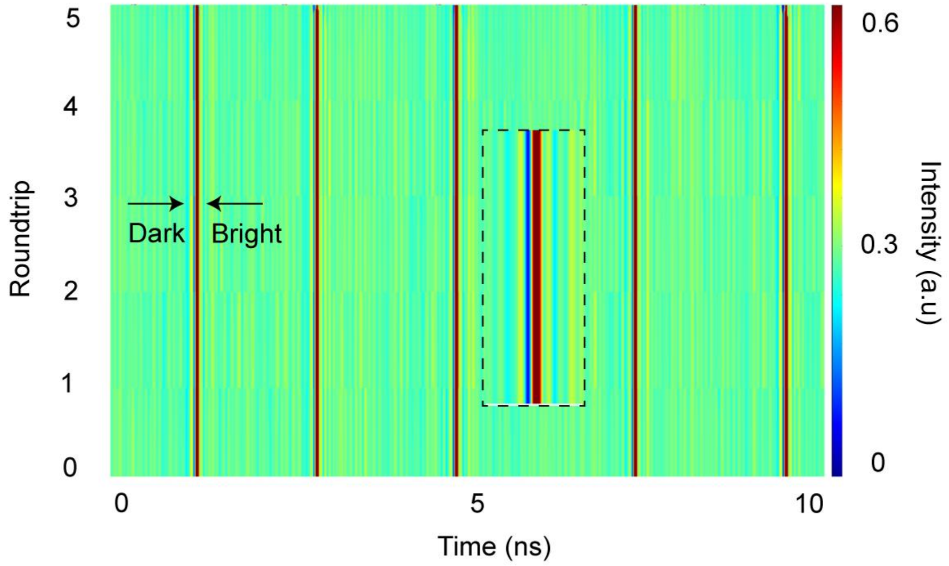

Fig. S4 Experimentally measured unequally spaced dark-bright soliton molecule state. Inset: Zoom in of one dark-bright soliton molecule.

Section 4: Simulation model and numerical technique used.

The light propagation in the cavity fibers is described by the coupled extended Ginzburg-Landau equations (CGLEs) [18],

$$\begin{aligned}\frac{\partial u}{\partial z} &= +\delta\frac{\partial u}{\partial t} - \frac{i\beta_{2u}}{2}\frac{\partial^2 u}{\partial t^2} + \frac{\beta_{3u}}{6}\frac{\partial^3 u}{\partial t^3} + i\gamma(|u|^2 + \frac{2}{3}|v|^2)u + \frac{g}{2}u + \frac{g}{2\Omega_g^{\,2}}\frac{\partial^2 u}{\partial t^2} \\ \frac{\partial v}{\partial z} &= -\delta\frac{\partial v}{\partial t} - \frac{i\beta_{2v}}{2}\frac{\partial^2 v}{\partial t^2} + \frac{\beta_{3v}}{6}\frac{\partial^3 v}{\partial t^3} + i\gamma(|v|^2 + \frac{2}{3}|u|^2)v + \frac{g}{2}v + \frac{g}{2\Omega_g^{\,2}}\frac{\partial^2 v}{\partial t^2}\end{aligned} \tag{4.1}$$

Where u and v are the normalized envelopes of the optical fields at different wavelengths. $\delta = \frac{1}{2}(\frac{1}{v_{gv}} - \frac{1}{v_{gu}})$ is the inverse group velocity difference between the modes. β_{2u} and β_{2v} are the second-order dispersion coefficients, β_{3u} and β_{3v} are the third-order dispersion (TOD) coefficients for the lights. γ represents the averaged nonlinear coefficient of the fiber, g is the saturable gain coefficient of the gain fiber and Ω_g is the gain bandwidth. For the light propagation in undoped fibers, $g = 0$. In our simulations, the gain saturation is described by

$$g=g_0 \exp[-\frac{\int (|u|^2+|v|^2)dt}{E_{sat}}] \quad (4.2)$$

Where g_0 is the small signal gain coefficient and E_{sat} is the saturation energy. We adopted a pulse tracing technique to simulate the laser operation. Briefly, we start with a certain initial light condition, and let the light circulate in the cavity. In different cavity fibers, we used the parameters of the individual fiber for the calculation. When the light meets the cavity output port, 10% of the light intensity is deducted from the light fields. After one cavity roundtrip, the light is then reinjected into the cavity as the input for the next round calculation. We used the standard split-step method to solve the coupled extended CGLEs (4.1). The numerical calculations were made on a 400 ps window and the periodic boundary condition was used. As mentioned, we also simulated the interactions between dark and bright solitons polarized along the same polarization axis by setting the XPM coupling coefficient σ in the E.q. (4.1) to 2,

$$\begin{aligned}\frac{\partial u}{\partial z} &= +\delta\frac{\partial u}{\partial t} - \frac{i\beta_{2u}}{2}\frac{\partial^2 u}{\partial t^2} + \frac{\beta_{3u}}{6}\frac{\partial^3 u}{\partial t^3} + i\gamma(|u|^2 + 2|v|^2)u + \frac{g}{2}u + \frac{g}{2\Omega_g^2}\frac{\partial^2 u}{\partial t^2} \\ \frac{\partial v}{\partial z} &= -\delta\frac{\partial v}{\partial t} - \frac{i\beta_{2v}}{2}\frac{\partial^2 v}{\partial t^2} + \frac{\beta_{3v}}{6}\frac{\partial^3 v}{\partial t^3} + i\gamma(|v|^2 + 2|u|^2)v + \frac{g}{2}v + \frac{g}{2\Omega_g^2}\frac{\partial^2 v}{\partial t^2}\end{aligned} \quad (4.3)$$

Noteworthy mentioning that although our current simulations are based on the CGLEs, under appropriate conditions, for example, if the gain bandwidth is larger than the soliton spectral bandwidth and the laser gain is balanced by the cavity losses, the CGLEs could be mathematically reduced to CNLSE [19].

Section 5: Simulation results on coexistence of dark and bright solitons.

Fig. S5 shows the initial pulse shape we used for the simulations on coexistence of dark and bright solitons along same polarization axis. The details of the simulation parameters for Fig. S6 are described in Tab. S1.

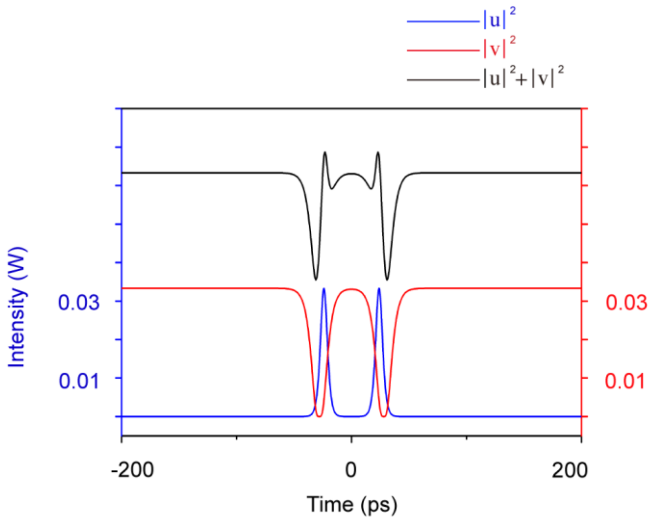

Fig. S5 Initially injected bright and dark pulses with a walk-off between them. Bule solid line: Initial pulse shape for the bright solitons. Red solid line: Initial pulse shape for the dark solitons. Black solid line: total intensity.

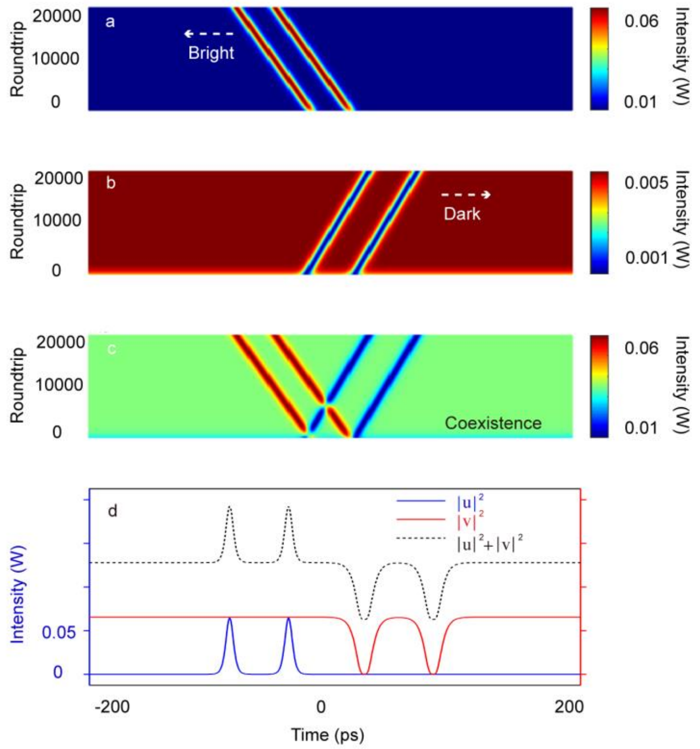

Fig. S6 Coexistence of dark and bright solitons along same polarization axis. a Evolution of the bright solitons along the horizontal polarization axis. **b** Evolution of the dark solitons along the vertical polarization axis. **c** Elastic collisions between the dark and bright solitons. **d** Red solid line: dark solitons observed at the last cavity round trip along the vertical polarization axis, blue solid line: bright solitons observed at the last cavity round trip along the horizontal polarization axis, black solid line: total laser emission at the last cavity round trip.

In the manuscript, we have numerically studied the soliton interactions between dark and bright solitons along the orthogonally polarized axis. Numerically, we also studied interactions between dark and bright solitons polarized along the same polarization direction. In this case the XPM coupling coefficient σ in Eq. (1) is changed to 2. We start the simulation with two weak bright and dark pulses. Each of the bright pulses has the $sech(A(t+dt))$ form, and each of the dark pulses has the $tanh(At)$ form. Here, dt represents an initial temporal separation between the dark and bright pulses. The bright solitons propagate in the net anomalous dispersion regime, while the dark solitons propagate in the net normal dispersion regime. Initially, they propagate in opposite directions in the cavity, as shown in Fig. S6. Under large group velocity mismatches, they travel independently in the cavity. The results coincide well with the experimental results, as shown in Fig. 6. We also numerically verified that if the coupling force between them is strong, the dark and bright solitons could be coupled with each other. Consequently, they could be trapped and co-propagate in the cavity, as shown in Supplementary Fig. S7.

Initial Pulse Shape	Averaged cavity parameters	SMF	EDF	DCF
Two dark and bright pulses with each in the form of<br><br>$u = sech(A(t+dt))$.<br>$v = tanh(At)$<br><br>$A = 1$;<br><br>*Initial temporal separation between dark and bright soliton*<br><br>$dt = 2$;	*Net averaged cavity dispersion:*<br><br>$\beta_{2v,ave} = -\beta_{2u,ave}$ $= 0.01\, ps^2\ km^{-1}$<br><br>$\beta_{3u,ave} = \beta_{3v,ave} =$ $0.001\, ps^3\ km^{-1}$<br><br>*Net averaged cavity birefringence:*<br><br>$\delta_{ave} = 0.05\ ps\ km^{-1}$<br><br>$\gamma$ =0.003 $(Wm)^{-1}$<br><br>Cavity loss: 10%	$\beta_{2u,SMF}$= -22.95 $ps^2\ km^{-1}$<br><br>$\beta_{2v,SMF}$= -22.93 $ps^2\ km^{-1}$<br><br>$L_B = 5\ km$<br><br>L=8.4 m	β_2= 63.4 $ps^2\ km^{-1}$<br><br>g_0 = 90 km^{-1}<br><br>Ω_g= 80 nm<br><br>E_s=0.001 nJ<br><br>$L_B = 5\ km$<br><br>L=3 m	β_2= 5.1 $ps^2\ km^{-1}$<br><br>$L_B = 5\ km$<br><br>L= 1.8 m

Tab. S1. Parameters used in the Fig. S6.

Section 6: Simulation on soliton trapping between dark and bright solitons.

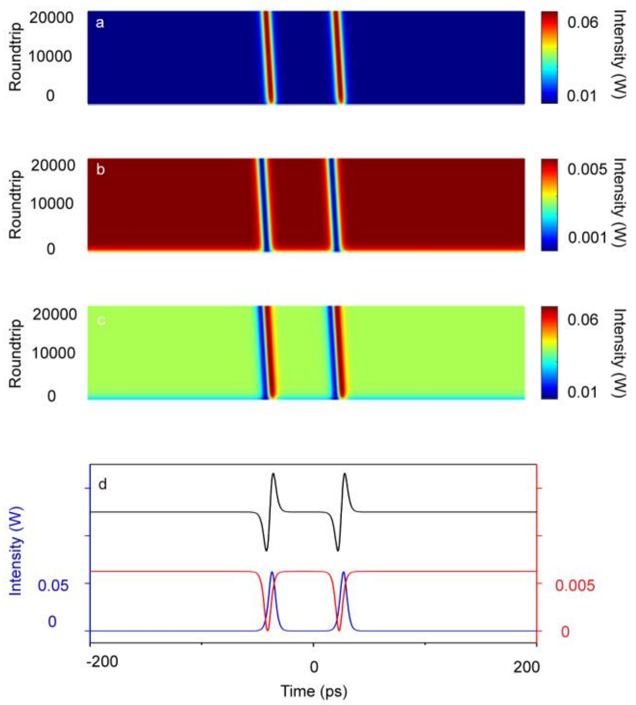

Fig. S7 Soliton trapping between dark and bright solitons. a Evolution of two bright solitons. **b** Evolution of two dark solitons. **c** Evolution of total intensity. **d** Red solid line: dark soliton at the last cavity round trip. Blue solid line: bright soliton at the last cavity round trip. Black solid line: total intensity of laser emission at the last cavity roundtrip.

Our simulation is based on E. q. (4.3). Similar to the case shown in Fig. S6. We also start the simulation with two weak bright and dark pulses. Each of the bright pulses has the form of $\mathrm{sech}(A(t+dt))$ and each of the dark pulse has the form of $\tanh(At)$. To propagate the dark and bright solitons at two sides of ZGVD point, we deliberately set the dispersion coefficient of the SMF for the dark and bright soliton as $\beta_{2v,SMF} = -22.93\, ps^2\, km^{-1}$ and $\beta_{2u,SMF} = -22.95\, ps^2\, km^{-1}$ respectively. Compared to the case shown in Fig. S6, we decreased the walk off between the dark and bright pulses by setting the beat length to be $L_B = 5\, km$, consequently, the group velocity mismatch reduced to $\delta = 0.0005\, ps\, km^{-1}$. With the smaller group velocity mismatch, the coupling between the dark and bright pulses become stronger, as a result, each of the dark soliton is captured by a bright soliton, they form a typical "1+1 soliton molecule" consisting of a scalar dark and a scalar bright soliton. Numerically we found that if δ is set as a negative value, corresponding to that the dark pulse travels faster than

the bright pulse, the formed bound solitons would be that the dark soliton leads the bright soliton. This result could be used to explain why two forms of (2+1) PSMs could be observed experimentally. Specifically, if the scalar dark soliton travels faster than the ODBS, the PSM will have the form as shown in Fig. 5a, while in the opposite condition, the PSM will feature those as shown in Fig. 5c.

Supplementary Videos

Visualization S1. Experimental demonstration on the coexistence of vector bright and vector dark solitons in a fiber laser. The video shows that the vector dark and vector bright solitons are simultaneously formed in the cavity. If one triggers the oscilloscope traces with the bright solitons, all the phase-locked bright solitons become frozen on the oscilloscope traces, while the vector dark solitons still move in the cavity suggesting that they are travelled at different group velocities.

Visualization S2. Experimental demonstration on the equally spaced bright solitons in a fiber laser. The video shows that stable bright solitons are formed in the cavity. Specifically, in the absence of unstable CW components or strong dispersive waves (DWs), the bright solitons are equally spaced in the cavity. If one triggers the oscilloscope traces with one bright soliton, all the bright solitons are frozen on the oscilloscope traces, no soliton wiggling is observed.

Visualization S3. Experimental demonstration on the unequally spaced bright solitons in a fiber laser. The video shows that with the presence of unstable CW components or strong DWs, the bright solitons start to move in the cavity. If one triggers the oscilloscope traces with one bright soliton, other bright solitons are still moving in the cavity with different speed, consequently, the bright soliton pattern becomes unequally spaced.

Visualization S4. Experimental demonstration on the bright soliton bunches in a fiber laser. The video shows that when the separations between solitons become smaller, a kind of bright soliton bunch could be formed as a result of long-range soliton interactions mediated by the DWs. If one triggers the oscilloscope traces with one bright soliton, other bright solitons within the bunch are still moving in the cavity with different speeds, consequently, the relative soliton positions constantly vary.

Supplementary References

1. Hu, X., Guo, J., Zhao, L. M., Li, L. & Tang, D. Y. Dissipative dark-bright vector solitons in fiber lasers. *Phys. Rev. A.* **101**, 063807 (2020).

2. Cundiff, S. T., Collings, B. C., Akhmediev, N., Soto-Crespo, J. M., Bergman, K. & Knox, H. Observation of Polarization-Locked Vector Solitons in an Optical Fiber. *Phys. Rev. Lett.* **82**, 3988 (1999).

3. Dianov, E. M., Luchnikov, A. V., Pilipetskii, A. N. & Prokhorov, A. M. Long-range interaction of picosecond solitons through excitation of acoustic waves in optical fibers. *Appl. Phys. B* **54**, 175-180 (1992).

4. Dianov, E. M., Luchnikov, A. V., Pilipetskii, A. N. & Starodumov, A. N. Electrostriction mechanism of soliton interaction in optical fibers. *Opt. Lett.* **15**, 6 (1990).

5. Pilipetskii, A. N., Golovchenko, E. A. & Menyuk, C. R. Acoustic effect in passive mode-locked fiber ring lasers. *Opt. Lett.* **20**, 8 (1995).

6. Kutz, J. N., Collings, B. C., Bergman, K. & Knox, W. H. Stabilized pulse spacing in soliton lasers due to gain depletion and recovery. *IEEE Journal of Quantum Electronics.* **34**, 9 (1998).

7. Grudinin, A. B. & Gray, S. Passive harmonic mode locking in soliton fiber lasers. *Opt. Soc. Am. B.* **14**, 1 (1997).

8. Socci, L. & Romagnoli, M. Long-range soliton interactions in periodically amplified fiber links. *Opt. Soc. Am. B.* **16**, 1 (1999).

9. Loh, W. H., Grudinin, A. B., Afanasjev, V. V. & Payne, D. N. Soliton interaction in the presence of a weak nonsoliton component. *Opt. Lett.* **19**, 10 (1994).

10. Sulimany, K. et al. Bidirectional soliton rain dynamics induced by casimir-like interactions in a graphene mode-locked fiber laser. *Phys. Rev. Lett.* **121**, 133902 (2018).

11. Rotschild, C., Alfassi, B., Cohen, O. & Segev, M. Long-range interactions between optical solitons. *Nature Physics.* **2**, 769-774 (2006).

12. Pang, M., He, W., Jiang, X. & Russell, P. St. J. All-optical bit storage in a fibre laser by optomechanically bound states of solitons. *Nature Photonics.* **10**, 454-458 (2016).

13. Nimmesgern, L. et al. Soliton molecules in femtosecond fiber lasers: universal binding mechanism and direct electronic control. *Optica.* **8**, 10 (2021).

14. Jang, J. K., Erkintalo, M., Murdoch, S. G. & Coen, S. Ultraweak long-range interactions of solitons observed over astronomical distances. *Nature Photonics.* **7**, 657-663 (2013).

15. Desurvire, E. Analysis of transient gain saturation and recovery in erbium-doped fiber amplifiers. *IEEE Photonics Technology Letters.* **1**, 8 (1989).

16. He, W., Pang, M., Yeh, D. H., Huang, J., Menyuk, C. R. & Russel, P. St. J. Formation of optical supramolecular structures in a fibre laser by tailoring long-range soliton interactions. *Nature Communications.* **10**, 5756 (2019).

17. He, W. B., Peng, M., Yeh, D. H., Huang, J. P. & Russell S. J. Synthesis and dissociation of soliton molecules in parallel optical-soliton reactors. *Light: Science & Applications.* **10**, 120 (2021).

18. Song, Y. F., Shi, X. J., Wu, C. F., Tang, D. Y. & Zhang, H. Recent progress of study on optical solitons in fiber lasers. *Appl. Phys. Rev.* **6**, 021313 (2019).

19. Kivshar, Y. S. et al. Optical Solitons: From Fibers to Photonic Crystals (Academic Press, 2003).
